# Omega-3 and omega-6 PUFAs induce the same GPR120-mediated signalling events, but with different kinetics and intensity in Caco-2 cells

**DOI:** 10.1186/1476-511X-12-101

**Published:** 2013-07-13

**Authors:** Kaia Mobraten, Trude M Haug, Charlotte R Kleiveland, Tor Lea

**Affiliations:** 1 Department of Chemistry, Biotechnology and Food Science, University of Life Sciences, Post box 5003, NO-1430 Aas, Norway; 2 Department of Biosciences, University of Oslo, NO-0316 Oslo, Norway; 3 Quality and Research Department, Ostfold Hospital Trust, NO-1603 Fredrikstad, Norway

**Keywords:** Polyunsaturated fatty acids (PUFAs), GPR120 Caco-2 cells

## Abstract

**Background:**

Omega-3 PUFAs are known to have anti-inflammatory properties, and different mechanisms are involved. GPR120 is a G-protein coupled receptor that has recently received attention because of its anti-inflammatory signalling properties after binding omega-3 PUFAs. However, both omega-3 and omega-6 PUFAs are natural GPR120 ligands. The aim of this study was to study possible differences in GPR120-mediated signalling events after treatment with different long-chain PUFAs in intestinal epithelial cells. We also investigated possible GPR120-mediated anti-inflammatory effects of different long-chain PUFAs that may be relevant in the understanding of how dietary PUFAs influence inflammatory responses in inflammatory diseases such as IBD.

**Methods:**

We used Caco-2 cells as a model system to study GPR120-mediated signalling events because we found this cell line to express GPR120, but not GPR40, another plasma membrane receptor for medium- and long chain fatty acids. Increase in cytosolic Ca^2+^concentration, activation of MAP kinase ERK1/2 and the inhibition of IL-1β induced NF-κB activity were studied to reveal potential differences in the activation of GPR120 by the omega-3 PUFAs eicosapentaenoic acid (EPA) and docosahexaenoic acid (DHA) and the omega-6 PUFA arachidonic acid (AA).

**Results:**

We found that EPA, DHA and AA enhanced the cytosolic concentration of the second messenger Ca^2+^ with the same efficiency, but with different kinetics. Both omega-3 and omega-6 PUFAs activated MAP kinase ERK1/2, but differences regarding kinetics and intensity were also observed in this pathway. ERK1/2 activation was shown to be dependent upon EGFR and Raf-1. We further investigated the ability of EPA, DHA and AA to inhibit NF-κB activity in Caco-2 cells. All PUFAs tested were able to inhibit IL-1β induced breakdown of IκBα after binding to GPR120, but with different potency.

**Conclusions:**

Our results show that EPA, DHA and AA elicit the same signalling events, but with different kinetics and efficiency through GPR120 in Caco-2 cells. We show, for the first time, that both omega-3 and omega-6 PUFAs inhibit NF-κB activation in intestinal epithelial cells. Our results may be important for understanding how dietary PUFAs influence inflammatory processes relevant in delineating effects of PUFAs in the treatment of IBD.

## Introduction

During digestion, dietary triglycerides are cleaved into monoglycerides and free fatty acids (FFAs). These FFAs are important nutrients in the daily energy intake, but they also act as signalling molecules mediating their effects through both nuclear- and plasma membrane receptors. Four FFA-binding G-protein coupled receptors (GPCRs) have been characterized, which bind FFAs with different chain lengths. FFAR2 (GPR43) and FFAR3 (GPR41) are activated by short-chain fatty acids (SCFAs), mainly acetate, propionate and butyrate, produced in colon by bacterial fermentation of indigestible carbohydrates [1,2]. GPR120 together with GPR40 (FFAR1) are activated by saturated and unsaturated medium- (C6-C12) and long-chain (C14-24) FFAs [3-6]. GPR40 is most abundantly expressed in pancreatic β-cells where it contributes to the regulation of insulin secretion [4,7,8]. GPR40 and GPR120 are both expressed in the gastrointestinal tract, where they regulate digestion through the secretion of cholecystokinin (CCK). In addition, they aid in insulin secretion by stimulating the release of the incretin hormones, glucagon-like peptide 1 (GLP-1) and glucose-dependent insulinotropic polypeptide (GIP) [4,6,9,10].

Polyunsaturated fatty acids (PUFAs) are principally divided into the omega-6 and the omega-3 families. Linoleic acid (LA) and arachidonic acid (AA) are essential omega-6 fatty acids and α-linolenic acid (ALA), eicosapentaenoic acid (EPA) and docosahexaenoic acid (DHA) are essential omega-3 fatty acids. Omega-3 fatty acids are generally considered anti-inflammatory, and different mechanisms are described. Incorporation of omega-3 fatty acids in the cell membrane lowers the content of AA, and thereby the substrate for synthesis of AA-derived eicosanoids, which are generally considered more pro-inflammatory than eicosanoids derived from EPA.

The transcription factor NF-κB is a key regulator of inflammation, and activation of NF-κB plays a key role in the initiation and perpetuation of the inflammatory diseases, including Crohn’s disease and Ulcerative colitis, collectively named inflammatory bowel diseases (IBDs). Omega-3 fatty acids reduce NF-κB mediated inflammatory signals, an effect that has been linked to the activation of peroxisome proliferator activated receptor -γ (PPAR-γ) and GPR120 [11-13]. Because of its anti-inflammatory properties, dietary supplementation of omega-3 PUFAs has been recommended in the treatment of patients with IBD. Animal studies have demonstrated beneficial effects from omega-3 PUFAs in the treatment of IBD [14-19], but results from human clinical trials are inconsistent with no clear evidence of efficacy [20]. However, *in vitro* studies have shown that omega-3 PUFAs exert anti-inflammatory effects in intestinal epithelial cells [21,22]. Thus, there is a need to know more about how omega-3 PUFAs interact with intestinal epithelial cells in order to understand how omega-3 PUFAs may have beneficial effects and to optimize the use of omega-3 PUFAs, in the treatment of IBD.

Omega-3 and omega-6 PUFAs are both natural ligands of GPR120, but recent review articles refer to its anti-inflammatory properties upon binding omega-3 PUFAs only [23,24]. GPR120 is expressed in the gastrointestinal tract, and the aim of this study was to elucidate signalling pathways activated by omega-3 and omega-6 PUFAs in intestinal epithelial cells. Caco-2 cells were used as a model system to study GPR120 signalling in intestinal epithelial cells. We further investigated the ability of both omega-3 and omega-6 PUFAs to inhibit NF-κB activation after binding to GPR120 on Caco-2 cells.

## Results

### PCR

**Figure 1:**
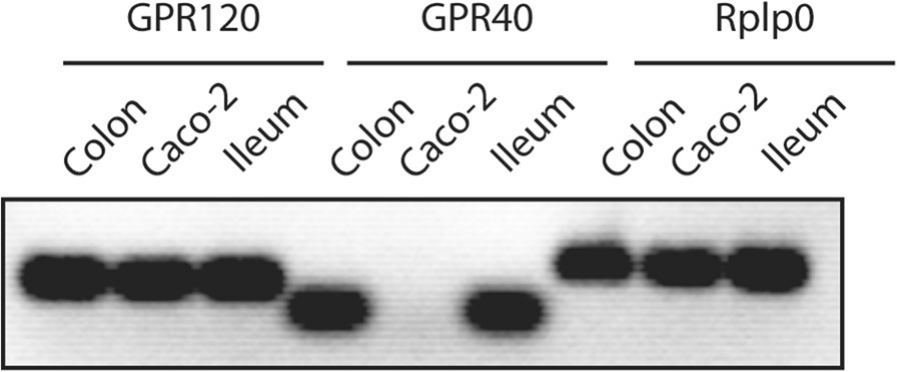
**Detection of GPR120 and GPR40 mRNA using PCR.** Total mRNA was isolated from tissue samples and Caco-2 cells using Qiagen RNase Mini Kit. Rplp0 is a housekeeping gene, and was used as a control. Each sample was analyzed in duplicates. The PCR products were visualised using 1% agarose gel electrophoresis.

PUFAs are natural ligands of GPR120 and GPR40. mRNA for GPR120 was detected in normal tissue both from ileum and colon, and in the Caco-2 cell line (Figure 1). On the other hand, GPR40 was only detected in the ileum and colon, which made Caco-2 cells a suitable system to specifically study PUFA-induced activation of GPR120.

### PUFAs elevate the cytosolic Ca^2+^ concentration

Studies describing GPR120 signalling in different cellular systems, including intestinal epithelial cells, have revealed that GPR120 is coupled to G_αq_[6,10,25]. However, GPR120 has not been described in Caco-2 cells previously. Since [Ca^2+^]_i_ can be used as a measure of G_αq_ activity, we investigated whether stimulation with EPA, DHA and AA was able to enhance [Ca^2+^]_i_ in Caco-2 cells.

**Figure 2:**
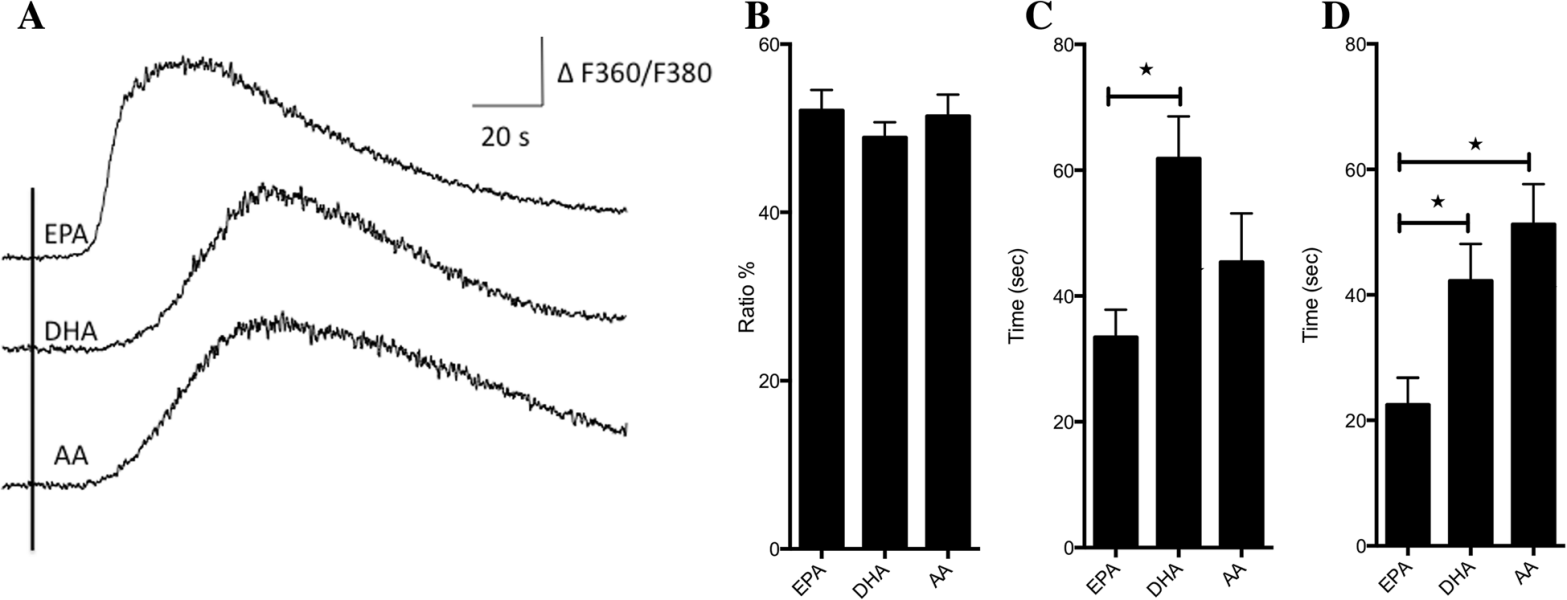
**Effect of PUFAs on [Ca**^**2+**^**]**_**i **_**in Caco-2 cells. (A)** Traces represent the effect of treating Caco-2 cells with 200 μM EPA, DHA and AA. F360/F380 is the ratio between emissions at the two different excitation wavelengths and reflects [Ca^2+^]_i_. PUFAs were applied for 10–30 sec from the time indicated by the vertical line. **(B)** The maximum [Ca^2+^]_i_ response obtained after treatment with the different PUFAs, estimated as % increase above baseline. **(C)** The time to peak of [Ca^2+^]_i_, measured as the time between 10% and 90% of maximum increase of [Ca^2+^]_i_. **(D)** The delay in seconds from start of treatment with indicated PUFAs until the increase of [Ca^2+^]_i_ had reached 10% of maximum [Ca^2+^]_i_. Data is presented as mean ± SEM from *n* = 15 (EPA), 14 (DHA) and 10 (AA) cells.

Figure 2 shows that treatment with 200 μM EPA, DHA or AA transiently increased [Ca^2+^]_i_ in Caco-2 cells. During treatment with the different PUFAs, the maximum increase of F360/F380, which monitors [Ca^2+^]_i_ were 52.2 ± 2.4% compared to untreated cells with EPA, 48.9 ± 1.8% with DHA, and 51.5 ± 2.6% with AA, and there were no significant differences between the responses to the three fatty acids (Figure 2B). However, Figure 2C shows that the time to peak (defined as time from 10% to 90% of max F360/F380 ratio increase) of [Ca^2+^]_i_ during treatment with 200 μM EPA was 33.4 ± 4.4 sec, and significantly (P < 0.05) faster than the time to peak of [Ca^2+^]_i_ after treatment with 200 μM DHA which was 61.8 ± 6.7 sec. The time to peak of [Ca^2+^]_i_ after treatment with 200 μM AA was 45.4 ± 7.7 sec, and was not significantly different from the time to peak of [Ca^2+^]_i_ after treatment with 200 μM DHA or EPA.

The time from the start of treatment with the different PUFAs, until the increase in [Ca^2+^]_i_ started (defined as time from start of treatment to 10% of max F360/F380 ratio increase), was also measured. Figure 2D shows that the delay after treatment 200 μM EPA was 22.5 ± 4.3 sec, and significantly shorter compared to treatment with both 200 μM DHA and 200 μM AA. The delay after treatment with 200 μM DHA and AA were 42.2 ± 5.9 sec and 51.2 ± 6.4 sec respectively.

### Activation of MAP kinase ERK1/2

**Figure 3:**
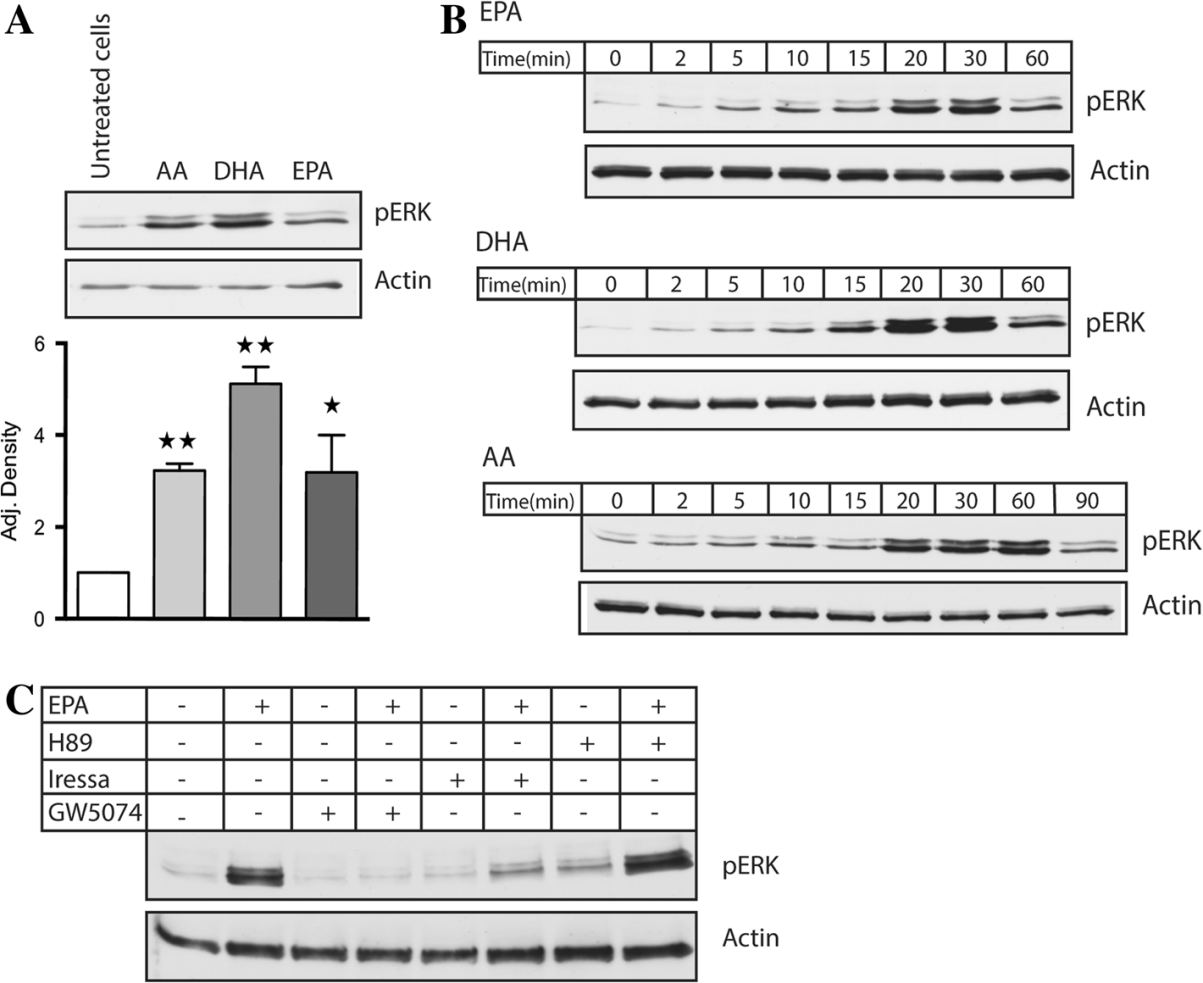
**The effect of PUFAs on ERK1/2 activation in Caco-2 cells. (A)** Cells were treated with 100 μM EPA, DHA and AA for 20 min. Differences in pERK 1/2 activity were determined using densitometry compared to untreated cells (n = 3, * p < 0.05, ** p < 0.01). **(B)** Cells were treated with 100 μM EPA and DHA from 0–60 min, and 100 μM AA from 0–90 min. **(C)** Cells were pretreated for 1 h with 10 μM H89, 10 μM GW5074 and 100 nM Iressa before treatment with 100 μM EPA for 20 min. The lysates were subjected to Western blotting to detect phosphorylated ERK1/2 and total actin. The figure is representative for at least three experiments with similar results.

Since differences were observed in the ability of the PUFAs to enhance cytosolic Ca^2+^- levels, we wanted to investigate whether such differences could also be detected in other signalling pathways. GPR120 is known to activate MAP kinase ERK1/2 in different cellular systems [6,11,26], and we therefore investigated if triggering of GPR120 with EPA, DHA or AA would activate ERK1/2 in Caco-2 cells. Figure 3A shows that treatment with 100 μM EPA, DHA or AA in 20 min enhanced ERK1/2 activity significantly (p < 0.5) in Caco-2 cells. Interestingly, the activation induced by 100 μM DHA at 20 min was stronger compared to treatment with EPA and AA, although not statistically significant using densitometry. Treatment with 100 μM EPA or DHA resulted in similar kinetics in the activation of ERK1/2, while AA gave a slower response (Figure 3B).

GPCRs can activate MAP kinase ERK1/2 through different mechanisms. To scrutinize possible pathways involved in ERK1/2 activation induced by EPA, we used the PKA inhibitor H89, the Raf-1 inhibitor GW5074 and the EGF receptor inhibitor Gifitinib “Iressa”. Pretreatment with 10 μM H89 for 1 h before treatment with 100 μM EPA for 20 min, did not affect ERK1/2 activation. However, pretreatment with 10 μM GW5074 and 100 nM Iressa 1 h before treatment with 100 μM EPA for 20 min, abolished ERK1/2 activity compared to treatment with 100 μM EPA alone (Figure 3C).

### Anti-inflammatory properties of PUFAs

**Figure 4:**
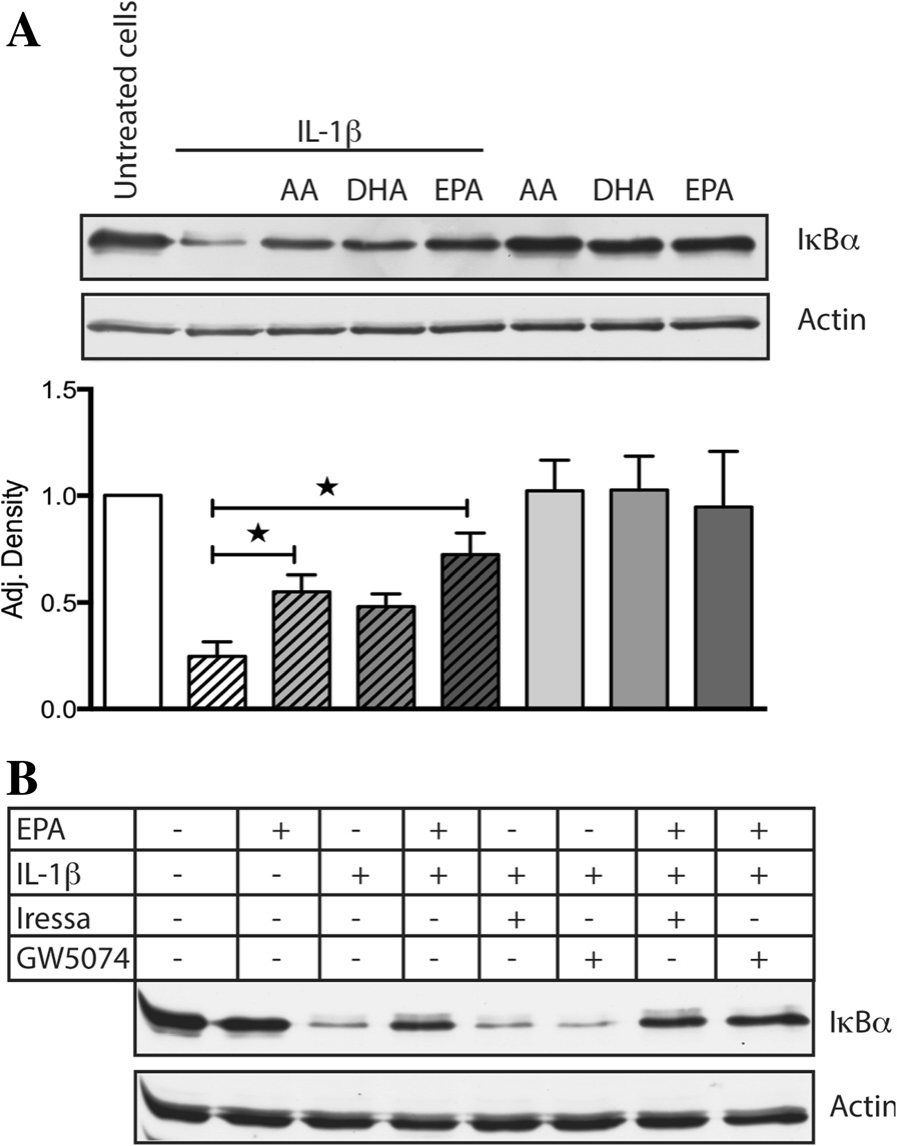
**The effect of PUFAs on IL-1β induced degradation of IκBα in Caco-2 cells. (A)** Cells were treated with 100 μM EPA, DHA, AA for 1 h, or with 100 ng/ml IL-1β for 30 min, or with 100 μM EPA, DHA, AA for 1 hour and then treated with 100 ng/ml IL-1β for 30 min. Differences in IκBα expression were determined using densitometry and compared to untreated cells (n = 3, * p < 0.05). **(B)** Cells were pretreated with 10 μM GW5074 and 100 nM Iressa for 1 h before treatment with 100 μM EPA for 1 h and then treated with 100 ng/ml IL-1β for 30 min. Cells were also treated with only 100 μM EPA, 10 μM GW5074 and 100 nM Iressa for 1 h and 100 ng/ml IL-1β for 30 min. The lysates were subjected to Western blotting to detect total IκBα and actin. The figure is representative for at least three experiments with similar results.

GPR120 has been shown to inhibit NF-κB signalling induced by lipopolysaccharide (LPS) after binding to Toll-like receptor 4 (TLR4) on macrophages and adipocytes, due to binding of β-arrestin2 in complex with transforming growth factor-β-activated kinase-1 (TAK1) and TAK1 binding protein-1 (TAB1) [11]. TLR4 and IL-1 receptor are members of the same superfamily and they use the same adaptors and kinases in their signalling pathways which lead to activation of NF-κB [27]. Since IL-1β is known to activate NF-κB signalling in Caco-2 cells [28], we investigated whether activation of GPR120 would influence IL-1β induced NF-κB activity in these cells. Treatment with 10 ng/ml IL-1β for 30 min decreased IκBα expression in Caco-2 cells compared to untreated cells. Stimulation with EPA or AA, but not DHA, inhibited IL-1β induced breakdown of IκBα significantly (p < 0.05) compared to treatment with IL-1β alone. Even though the PUFAs inhibited IL-1β induced breakdown of IκBα with different efficiencies, these differences were not considered significant compared to each other (Figure 4A).

Since activation of MAP kinase ERK1/2 was dependent upon EGF receptor transactivation and Raf-1 activation, we tested whether these were involved in the ability of GPR120 to inhibit IL-1β induced breakdown of IκBα. Figure 4B shows that neither Raf-1 nor the EGF receptor are involved in the ability of EPA to inhibit IL-1β induced breakdown of IκBα.

## Discussion

Results from this study show that triggering of GPR120 with EPA, DHA or AA in Caco-2 cells activated three independent intracellular signalling events; accumulation of cytosolic Ca^2+^, EGF receptor- and Raf-1-dependent activation of MAP kinase ERK1/2, and EGF receptor- and Raf-1-independent inhibition of IL-1β induced NF-κB activation. Interestingly, EPA, DHA and AA were able to activate these pathways with different kinetics and intensity.

The finding that GPR120 activates G_αq_ and the subsequent increase of cytosolic Ca^2+^ are in agreement with previous studies in other cellular systems [6,11,25,29]. In the first study to describe induction of GPR120 signalling by FFAs, EPA and DHA were shown to have almost equal ability to enhance [Ca^2+^]_i_ in HEK293 cells transfected with GPR120 [6]. Oh et al. (2010) used GPR120-transfected HEK293 cells to study accumulation of cytosolic Ca^2+^ after treatment with FFAs, and found that EPA and DHA enhanced [Ca^2+^]_i_ with same intensity. However, AA did not enhance [Ca^2+^]_i_ in this study [11]. We found that EPA, DHA and AA were all able to enhance [Ca^2+^]_i_ with the same efficiency, but with different kinetics. The finding that EPA was able to enhance [Ca^2+^]_i_ faster than DHA and AA may be due to differences in ligand affinity to GPR120 on Caco-2 cells.

The differences in Ca^2+^ signalling dynamics induced by the different PUFAs, did not correlate with the activation of pERK 1/2 induced by the same PUFAs. AA induced a slower pERK 1/2 kinetic response than EPA and DHA. DHA gave strongest pERK 1/2 activation after 20 min of treatment compared to AA and EPA, but these differences were not considered significant. We further show that the activation of pERK 1/2 is a result of EGF receptor transactivation involving Raf-1 kinase. Long-chain PUFAs are known to activate EGF receptor in other cellular systems [30,31], but this activation has not been linked to GPR120. In the present study, we show that pretreatment with the EGF receptor specific inhibitor Iressa, and Raf-1 inhibitor GW5074 abolish ERK1/2 activation induced by EPA. Raf-1 is a crucial kinase in the MAP kinase signalling cascade leading to activation of ERK1/2. EGF receptor can activate the Ras-Raf-Mek cascade through the activation of Grb2 and SOS. The finding that PKA inhibitor H89 did not influence ERK1/2 activity is in line with previous studies describing GPR120 signalling, where no study has reported activation of PKA after GPR120 activation.

Both cytosolic Ca^2+^ increase and pERK1/2 activation are involved in GPR120-induced secretion of GLP-1 and CCK from intestinal cells [6,10,32,33]. The findings that these pathways are activated differently by EPA, DHA and AA in Caco-2 cells might gain new insights into variations in the different PUFAs ability to induce the secretion of GLP-1 and CCK.

Increased dietary intake of omega-6 PUFAs, and a lower dietary intake of omega-3 PUFAs are though to contribute to the development of IBD [34,35]. There is clear evidence that omega-6 PUFAs exert pro-inflammatory events compared to omega-3 PUFAs [34]. However, our results suggest that both omega-3 and omega-6 PUFAs exert the same anti-inflammatory effects by inhibiting NF-κB activity after binding GPR120 on Caco-2 cells. DHA, EPA and AA were all able to inhibit IL-1β induced breakdown of IκBα, but EPA and AA mediated the strongest inhibitory effects. Since this effect was independent of Raf-1 and EGF receptor, we speculate whether GPR120 may inhibit IL-1β signalling in the same manner as TLR4 signalling. These findings challenge the view that only omega-3 PUFAs exert anti-inflammatory effects after binding GPR120. Our results may also be important in the understanding of how long-chain PUFAs, not only omega-3 PUFAs influence the inflammatory response in patients with IBD.

In conclusion, our results show that both omega-3 and omega-6 PUFAs activate the same GPR120-mediated signalling events in Caco-2 cells, but differences regarding intensity and kinetics were observed. Previously, anti-inflammatory effects of GPR120 have only been linked to omega-3 PUFAs. We show, for the first time that they might be linked to both omega-3 and omega-6 PUFAs.

## Materials and methods

### Materials

Sodium salts of arachidonic acid, docosahexaenoic acid and eicosapentaenoic acid were from Sigma-Aldrich. Antibodies targeted against Phospho-p44/42 MAPK (ERK1/2) and IκBα were from Cell Signaling Technology and the antibody targeted against α-actin was from Sigma-Aldrich. HRP-conjugated secondary antibodies, Goat Anti-Mouse IgG (H+L) and Goat Anti-Rabbit IgG (H+L) were from Southern Biotech. Other chemicals used were: H89 from Alexis Chemicals, GW5074 from Sigma-Aldrich and Gefinib “Iressa” from Selleck Chemicals LLC.

### Cell culture

The human colon adenocarcinoma cell line Caco-2 (ATTC; HTG-37^TM^) were grown in RPMI-1640 supplemented with 10% fetal calf serum (FCS), 100 μM non-essential amino acids, 1 mM sodium pyruvate (all from PAA Laboratories), and gentamicin (24 mg/l) (Lonza). Cells were maintained in a humidified incubator at 37°C and 5% CO_2_.

### PCR

RNA from both tissue and cells were isolated using Qiagen RNase Mini kit (Qiagen). Complementary DNA (cDNA) was synthesized with Super Script® Vilo^TM^ (Applied Biosystems) using 20 μl of reaction mixture containing 2 μg RNA. PCR was set up using TaqMan® Master Mix and TaqMan® probes: GPR120 (Hs00699184_m1), GPR40 (Hs03045166_s1) and rplp0 (Hs99999902_m1). 50 ng template was used in each reaction.

### Cytosolic Ca^2+^ measurements

Measurements of the cytosolic Ca^2+^ concentration were performed in the following extracellular solution (EC): 150 mM NaCl, 5 mM KCl, 2.4 mM CaCl_2_, 1.3 mM MgCl_2_, 10 mM glucose and 10 mM HEPES, adjusted to pH 7.4 by NaOH.

Approximately 10^6^ Caco-2 cells were seeded out in collagen-coated glass-bottom dishes (MatTek Coorporation) 3–4 days prior to experiment. Cells were loaded with 5 μM of the fluorescent Ca^2+^ indicator fura-2/AM (Molecular Probes) in EC for 45 min at 37°C, followed by washout of the fura-2 ester and further 30 min incubation at room temperature. Then, cells were mounted on an Olympus OSP-3 system for dual excitation fluorometry (Olympus). The excitation light was switched at 200 Hz between 360 and 380 nm using a rotating mirror. The emitted fluorescence was recorded at 510 nm with a photomultiplier, and the measurements were restricted to single cells by a pinhole diaphragm. The ratio between emissions at the two different excitation wavelengths (F360/F380) reflects the cytosolic Ca^2+^ concentration [Ca^2+^]_i_. In the present study, the relative increase in [Ca^2+^]_i_ is used as a measure of the response to the PUFAs. Therefore, calibration in order to determine the absolute Ca^2+^ concentrations was not performed. Cells were exposed to the different PUFAs by pressure ejection (about 1 kPa) from a micropipette placed about 40 μm from the cell. As negative control, cells were exposed to EC by pressure ejection using the same conditions as described above. No artefacts were observed when ejecting normal EC onto cells, and the increase in [Ca^2+^]_i_ was compared to untreated cells.

### Cell stimulation and lysis

Sodium salts of DHA, EPA and AA were dissolved in autoclaved deionized water and further diluted in RPMI 1640. 0.5 × 10^6^ cells were seeded per well in a 24-well plate. The day after, the cells were serum-starved for 24 h in RPMI-1640 supplemented with 1% FCS, and then stimulated as indicated. After stimulation, the cells were lysed in 100 μl lysis buffer (25 mM Tris, pH7.5, 1% NP-40, 100 mM NaCl, 20 mM NaF, 1 mM orthovanadate, 1 μg/ml leupeptin, 1 μg/ml antipain, 1 μg/ml pepstatin A and 1 μg/ml chymostatin )per well for 30 min at 4°C. Lysates were spun at 4°C for 15 min at 15 000 rpm, and the supernatants were used for Western blotting.

### Western blotting

Cell lysates were electrophoresed in 10% SDS-polyacrylamide gels. The separated proteins were transferred to PVDF membranes by Western blotting. Upon blotting, the membranes were blocked with 5% non-fat dry milk for 1 h, and probed with specific antibodies to detect protein of interest. Luminata^TM^ Crescendo Western HRP Substrate (Merck Millipore) was used as substrate for HRP-conjugated secondary antibodies, and the membranes were exposed using X-Ray film (Pierce). Densitometry analysis was performed using Image J 1.46r. Adj. density was obtained by comparing the intensity of the band of interest with the intensity of the actin band from the same sample.

## Competing interests

There are no conflicts of interest to declare.

## Authors’ contributions

K.M was involved in the experimental procedures, data analysis and writing of the manuscript. T.M.H was involved in the cytosolic Ca^2+^ measurements, and writing of the manuscript. C.R.K. and T.L contributed to the writing of the manuscript. T.L. supervised the project. All authors read and approved the final manuscript.

## References

[B1] Le Poul E Loison C Struyf S Springael JY Lannoy V Decobecq ME Brezillon S Dupriez V Vassart G Van Damme J Functional characterization of human receptors for short chain fatty acids and their role in polymorphonuclear cell activation J Biol Chem 2003 278 25481 25489 10.1074/jbc.M301403200 12711604

[B2] Nilsson NE Kotarsky K Owman C Olde B Identification of a free fatty acid receptor, FFA2R, expressed on leukocytes and activated by short-chain fatty acids Biochem Biophys Res Commun 2003 303 1047 1052 10.1016/S0006-291X(03)00488-1 12684041

[B3] Briscoe CP Tadayyon M Andrews JL Benson WG Chambers JK Eilert MM Ellis C Elshourbagy NA Goetz AS Minnick DT The orphan G protein-coupled receptor GPR40 is activated by medium and long chain fatty acids J Biol Chem 2003 278 11303 11311 10.1074/jbc.M211495200 12496284

[B4] Itoh Y Kawamata Y Harada M Kobayashi M Fujii R Fukusumi S Ogi K Hosoya M Tanaka Y Uejima H Free fatty acids regulate insulin secretion from pancreatic beta cells through GPR40 Nature 2003 422 173 176 10.1038/nature01478 12629551

[B5] Kotarsky K Nilsson NE Flodgren E Owman C Olde B A human cell surface receptor activated by free fatty acids and thiazolidinedione drugs Biochem Biophys Res Commun 2003 301 406 410 10.1016/S0006-291X(02)03064-4 12565875

[B6] Hirasawa A Tsumaya K Awaji T Katsuma S Adachi T Yamada M Sugimoto Y Miyazaki S Tsujimoto G Free fatty acids regulate gut incretin glucagon-like peptide-1 secretion through GPR120 nature medicine 2005 11 90 94 10.1038/nm1168 15619630

[B7] Latour MG Alquier T Oseid E Tremblay C Jetton TL Luo J Lin DC Poitout V GPR40 is necessary but not sufficient for fatty acid stimulation of insulin secretion in vivo Diabetes 2007 56 1087 1094 10.2337/db06-1532 17395749 PMC1853382

[B8] Steneberg P Rubins N Bartoov-Shifman R Walker MD Edlund H The FFA receptor GPR40 links hyperinsulinemia, hepatic steatosis, and impaired glucose homeostasis in mouse Cell Metab 2005 1 245 258 10.1016/j.cmet.2005.03.007 16054069

[B9] Edfalk S Steneberg P Edlund H Gpr40 is expressed in enteroendocrine cells and mediates free fatty acid stimulation of incretin secretion Diabetes 2008 57 2280 2287 10.2337/db08-0307 18519800 PMC2518478

[B10] Tanaka T Katsuma S Adachi T Koshimizu TA Hirasawa A Tsujimoto G Free fatty acids induce cholecystokinin secretion through GPR120 Naunyn Schmiedebergs Arch Pharmacol 2008 377 523 527 10.1007/s00210-007-0200-8 17972064

[B11] Oh DY Talukdar S Bae EJ Imamura T Moringa H Fan W Li P Lu WJ Watkins SM Olefsky JM GPR120 is an Omega-3 fatty acid receptor mediating potent anti-inflammatory and insulin-sensitizing effects Cell 2010 142 687 698 10.1016/j.cell.2010.07.041 20813258 PMC2956412

[B12] Zapata-Gonzalez F Rueda F Petriz J Domingo P Villarroya F Diaz-Delfin J de Madariaga MA Domingo JC Human dendritic cell activities are modulated by the omega-3 fatty acid, docosahexaenoic acid, mainly through PPAR(gamma):RXR heterodimers: comparison with other polyunsaturated fatty acids J Leukoc Biol 2008 84 1172 1182 10.1189/jlb.1007688 18632990

[B13] Kong W Yen JH Vassiliou E Adhikary S Toscano MG Ganea D Docosahexaenoic acid prevents dendritic cell maturation and in vitro and in vivo expression of the IL-12 cytokine family Lipids Health Dis 2010 9 12 10.1186/1476-511X-9-12 20122166 PMC2827414

[B14] Vilaseca J Salas A Guarner F Rodriguez R Martinez M Malagelada JR Dietary fish oil reduces progression of chronic inflammatory lesions in a rat model of granulomatous colitis Gut 1990 31 539 544 10.1136/gut.31.5.539 2161781 PMC1378570

[B15] Nieto N Torres MI Rios A Gil A Dietary polyunsaturated fatty acids improve histological and biochemical alterations in rats with experimental ulcerative colitis J Nutr 2002 132 11 19 11773501 10.1093/jn/132.1.11

[B16] Hudert CA Weylandt KH Lu Y Wang J Hong S Dignass A Serhan CN Kang JX Transgenic mice rich in endogenous omega-3 fatty acids are protected from colitis Proc Natl Acad Sci U S A 2006 103 11276 11281 10.1073/pnas.0601280103 16847262 PMC1544078

[B17] Shoda R Matsueda K Yamato S Umeda N Therapeutic efficacy of N-3 polyunsaturated fatty acid in experimental Crohn's disease J Gastroenterol 1995 30 Suppl 8 98 101 8563904

[B18] Yuceyar H Ozutemiz O Huseyinov A Saruc M Alkanat M Bor S Coker I Batur Y Is administration of n-3 fatty acids by mucosal enema protective against trinitrobenzene-induced colitis in rats? Prostaglandins Leukot Essent Fatty Acids 1999 61 339 345 10.1054/plef.1999.0111 10718105

[B19] Andoh A Tsujikawa T Ishizuka I Araki Y Sasaki M Koyama S Fujiyama Y N-3 fatty acid-rich diet prevents early response of interleukin-6 elevation in trinitrobenzene sulfonic acid-induced enteritis Int J Mol Med 2003 12 721 725 14533000

[B20] Cabre E Manosa M Gassull MA Omega-3 fatty acids and inflammatory bowel diseases - a systematic review Br J Nutr 2012 107 Suppl 2 S240 252 22591898 10.1017/S0007114512001626

[B21] Ramakers JD Mensink RP Schaart G Plat J Arachidonic acid but not eicosapentaenoic acid (EPA) and oleic acid activates NF-kappaB and elevates ICAM-1 expression in Caco-2 cells Lipids 2007 42 687 698 10.1007/s11745-007-3071-3 17610002 PMC2039812

[B22] Marion-Letellier R Butler M Dechelotte P Playford RJ Ghosh S Comparison of cytokine modulation by natural peroxisome proliferator-activated receptor gamma ligands with synthetic ligands in intestinal-like Caco-2 cells and human dendritic cells–potential for dietary modulation of peroxisome proliferator-activated receptor gamma in intestinal inflammation Am J Clin Nutr 2008 87 939 948 18400717 10.1093/ajcn/87.4.939

[B23] Im DS Omega-3 fatty acids in anti-inflammation (pro-resolution) and GPCRs Prog Lipid Res 2012 51 232 237 10.1016/j.plipres.2012.02.003 22542696

[B24] Osborn O Olefsky JM The cellular and signaling networks linking the immune system and metabolism in disease Nat Med 2012 18 363 374 10.1038/nm.2627 22395709

[B25] Tanaka T Yano T Adachi T Koshimizu TA Hirasawa A Tsujimoto G Cloning and characterization of the rat free fatty acid receptor GPR120: in vivo effect of the natural ligand on GLP-1 secretion and proliferation of pancreatic beta cells Naunyn Schmiedebergs Arch Pharmacol 2008 377 515 522 10.1007/s00210-007-0250-y 18320172

[B26] Katsuma S Hatae N Yano T Ruike Y Kimura M Hirasawa A Tsujimoto G Free fatty acids inhibit serum deprivation-induced apoptosis through GPR120 in a murine enteroendocrine cell line STC-1 J Biol Chem 2005 280 19507 19515 10.1074/jbc.M412385200 15774482

[B27] Medzhitov R Toll-like receptors and innate immunity Nat Rev Immunol 2001 1 135 145 10.1038/35100529 11905821

[B28] Al-Sadi R Ye D Said HM Ma TY IL-1beta-induced increase in intestinal epithelial tight junction permeability is mediated by MEKK-1 activation of canonical NF-kappaB pathway Am J Pathol 2010 177 2310 2322 10.2353/ajpath.2010.100371 21048223 PMC2966790

[B29] Hara T Hirasawa A Sun Q Sadakane K Itsubo C Iga T Adachi T Koshimizu TA Hashimoto T Asakawa Y Tsujimoto G Novel selective ligands for free fatty acid receptors GPR120 and GPR40 Naunyn Schmiedebergs Arch Pharmacol 2009 380 247 255 10.1007/s00210-009-0425-9 19471906

[B30] Soto-Guzman A Robledo T Lopez-Perez M Salazar EP Oleic acid induces ERK1/2 activation and AP-1 DNA binding activity through a mechanism involving Src kinase and EGFR transactivation in breast cancer cells Mol Cell Endocrinol 2008 294 81 91 10.1016/j.mce.2008.08.003 18775472

[B31] Vacaresse N Lajoie-Mazenc I Auge N Suc I Frisach MF Salvayre R Negre-Salvayre A Activation of epithelial growth factor receptor pathway by unsaturated fatty acids Circ Res 1999 85 892 899 10.1161/01.RES.85.10.892 10559135

[B32] Adachi T Tanaka T Takemoto K Koshimizu TA Hirasawa A Tsujimoto G Free fatty acids administered into the colon promote the secretion of glucagon-like peptide-1 and insulin Biochem Biophys Res Commun 2006 340 332 337 10.1016/j.bbrc.2005.11.162 16356474

[B33] Shah BP Liu P Yu T Hansen DR Gilbertson TA TRPM5 is critical for linoleic acid-induced CCK secretion from the enteroendocrine cell line, STC-1 Am J Physiol Cell Physiol 2012 302 C210 219 10.1152/ajpcell.00209.2011 21998136 PMC3328913

[B34] Simopoulos AP The importance of the ratio of omega-6/omega-3 essential fatty acids Biomed Pharmacother 2002 56 365 379 10.1016/S0753-3322(02)00253-6 12442909

[B35] Shoda R Matsueda K Yamato S Umeda N Epidemiologic analysis of Crohn disease in Japan: increased dietary intake of n-6 polyunsaturated fatty acids and animal protein relates to the increased incidence of Crohn disease in Japan Am J Clin Nutr 1996 63 741 745 8615358 10.1093/ajcn/63.5.741

